# Comparison of two validated oscillometric devices in a home-like setup reveals pronounced blood pressure differences and reduced precision

**DOI:** 10.1038/s41440-025-02514-3

**Published:** 2026-01-09

**Authors:** Joachim Zahnd, Brian Thompson, Pierre-Antonin Rigon, Patrick Taffé, Gregoire Wuerzner

**Affiliations:** 1 Service of nephrology and hypertension, Lausanne University Hospital and University of Lausanne, Lausanne, Switzerland; 2 Center for Primary Care and Public Health (Unisanté), Division of Biostatistics, University of Lausanne, Lausanne, Switzerland

**Keywords:** Home blood pressure monitoring, Self-blood pressure monitoring, Implemental hypertension

## Abstract

Home blood pressure monitoring (HBPM) is essential for long-term hypertension management, but its accuracy and reliability is questionable due to user inconsistencies and non-standard usage conditions. This study compared two validated HBPM devices – a wrist-based and a reference upper-arm monitor – in a home-like setup where participants placed the devices themselves. A total of 121 participants underwent four concurrent blood pressure (BP) measurements, with two taken in each arm, followed by a crossover of devices between arms after the initial two readings. Wrist-derived BP readings were higher than upper-arm measurements. With higher blood pressure levels, both devices exhibited greater bias, accompanied by reduced precision in systolic BP measurements. Additionally, hypertension classification showed only moderate agreement (Cohen’s kappa=0.58). The wrist monitor tended to over-diagnose hypertension and exhibited greater variability than the upper-arm device. These findings highlight the need for more guidance and education as well as critical evaluation of home blood pressure measurements provided by patients.

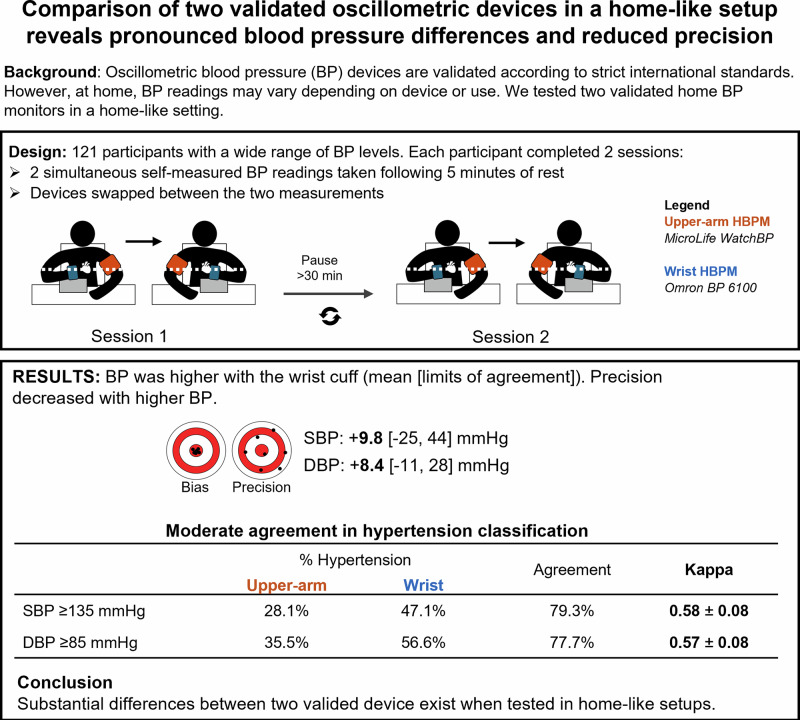

## Introduction

Hypertension diagnosis and management rely almost exclusively on blood pressure (BP) measurements performed with cuff-based oscillometric monitors. Among these, home BP monitoring (HBPM) is uniquely positioned to provide longitudinal data that has been associated with cardiovascular outcomes [1]. However, despite its potential and its recommendation by guidelines, HBPM is subject to measurement inaccuracies and to poor patients’ engagement on the long term [2–4]. These challenges are amplified in the home setting, where measurement conditions are typically less controlled. Healthcare providers have limited visibility into these conditions, which can lead to treatment decisions based on inaccurate or incomplete data.

To determine the impact of the setting on blood pressure readings and hypertension diagnosis, we conducted a nested study (NCT06368206) comparing two validated, cuff-based HBPM devices under conditions that mimic real-world use. We assessed systolic and diastolic BP (SBP, DBP) agreement between a wrist-worn device (Omron BP RS4, Omron Healthcare Co. Ltd, Japan) and a reference upper-arm monitor (Microlife WatchBP Home, Microlife, Switzerland) [5, 6]. Additionally, we assessed the precision and evaluated the agreement in hypertension classification between the two devices.

## Methods

This study was designed to replicate real-world, home-like conditions for HBPM in adult participants and was conducted in a dedicated outpatient consultation room. Although a healthcare professional was present for general supervision, their role was limited to ensuring participant safety and adherence to the measurement sequence, with little to no influence on the actual BP measurements. An adequately sized arm cuff (medium or large) were provided by the supervisors according to arm circumference. Participants with wrist circumferences >23 cm were excluded. Participants were solely responsible for placing the BP monitors according to the instruction manuals, just as if they had purchased the device from a store or pharmacy (Supplemental Fig. 1). While this approach does not align with guidelines, it was intentionally chosen to reflect real-world use, where users often set up and operate BP monitors without professional assistance. By adopting this methodology, we aimed to capture the variability and challenges of real-world HBPM use, including intrinsic measurement differences within devices, potential errors in device placement and operation that naturally occur in unsupervised home environments.

BP measurements were collected during two sessions, approximately one hour apart. In each session, the monitors were placed on opposite arms, and after measurement, they were swapped, ensuring that BP was measured simultaneously on both arms by both monitors across both sessions, resulting in a total of four measurements per participant. The average of the four measurements were used in the analyses. Participants with interarm differences of ≥15 mmHg SBP or ≥10 mmHg DBP during screening were excluded (BS EN ISO 81060-2:2019 + A1:2020). The readings from the wrist monitor were compared with the readings from the reference upper -arm device, here considered as the reference. Population-level differences in BP measurements between the two monitors were investigated using the conventional Bland-Altman Limits of Agreement (LoA) and recently proposed Taffé Biasplot methods [7, 8]. In the latter method, precision is expressed as the standard deviation of the predicted distribution for a predicted probability. The hypertension classification performance of both devices was compared using 135/85 mmHg as the threshold for home SBP and DBP hypertension, respectively [1]. Fisher’s exact test, agreement percentage, and Cohen’s kappa were calculated to assess and compare the classification of the two devices.

The study was approved by the local ethics committee (CER-VD) and conducted in accordance with the Declaration of Helsinki and the International Conference on Harmonisation Good Clinical Practice guidelines (ICH-GCP E6[R2]). Written informed consent was obtained from all participants prior to inclusion in the study.

## Results

A total of 132 adult participants across wide BP ranges were recruited. After exclusion of participants with interrupted visits (*N* = 9) or who did not receive the user manual (*N* = 2), 121 participants were included in the analysis. The participants were 48.5 ± 15.9 (mean and standard deviation) years old, 51.5% were female and their body mass index was 28.1 ± 5.5 kg/m².

**Fig. 1:**
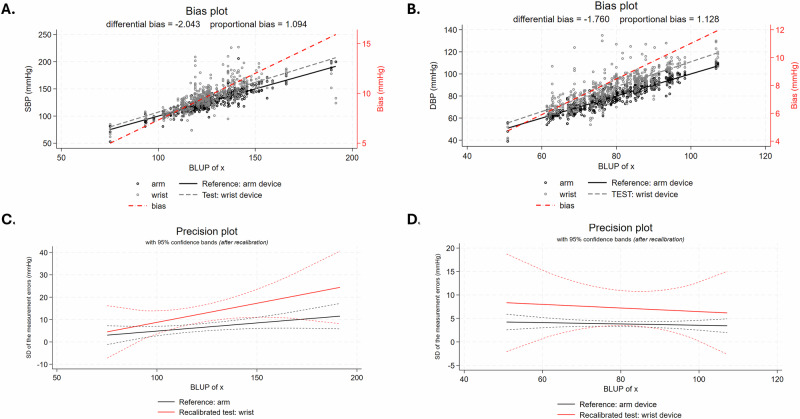
Bias plots (upper, **A**: SBP, **B**: DBP) and Precision plots (lower; **C**: SBP, **D**: DBP) comparing simultaneous BP measurements performed by oscillometric wrist and upper-arm monitors on opposing arms. The x-axis displays the “BLUP of x”, where BLUP stands for Best Linear Unbiased Prediction: an estimate of the true BP of the study participants given the two noisy measured values by the oscillometric devices. Both Bias plots show that the higher the true BP the larger the bias (shown by the red dash-dotted line measuring the vertical distance between the two regression lines (black for the upper-arm and grey for the wrist devices) whose distance can be read on the right y-axis). Likewise, the two Precision plots show that the standard deviation of measurement errors was larger for the wrist device. SBP systolic blood pressure, DBP diastolic blood pressure

The comparison between the wrist and upper-arm HBPM devices is shown in Fig. 1 and Supplemental Fig. 2 (using both the Bland-Altman LoA plot and Taffé Bias and Precision plots). The results indicate significant differences between the two devices, with mean biases exceeding generally accepted thresholds for clinical validation (Bland-Altman LoA plot: mean bias = 9.8 (SBP), lower and upper limits [−25, 44] mmHg; mean bias = 8.4 (DBP), lower and upper limits [−11, 28] mmHg). Of particular importance is the broad LoA for SBP, which suggests wrist-based measurements may deviate by up to 44 mmHg from the upper-arm reference for a given participant. In addition, Taffé Biasplot shows that the magnitude of bias was not constant but increased with higher BP values. While the bias remained approximately 5 mmHg for SBP around 75 mmHg and 5 mmHg for DBP around 55 mmHg, it increased to over 15 mmHg for SBP above 180 mmHg and exceeded 12 mmHg for DBP above 110 mmHg. Furthermore, on the Precision plot the upper-arm device demonstrated higher precision than the wrist device in both SBP and DBP, as indicated by the smaller standard deviation of measurement errors. This suggests that, in addition to exhibiting systematic differences, wrist-based BP measurements also show greater variability, a meaningful finding when it comes to clinical decision making. Finally, precision of systolic BP decreases with increasing BP for both devices.

The hypertension classification for each device is presented in Table 1. The wrist device tended to overdiagnose hypertension for both SBP and DBP compared to the reference arm device. Despite a statistically significant association between the classifications from both methods and an overall agreement above 75% for SBP and DBP, Cohen’s kappa revealed that the level of agreement was only moderate.

**Table 1:** Hypertension classification performed by both upper-arm and writs BP devices

	% Hypertension		Fisher’s test (*P*-value)	Agreement	Kappa
	Upper-arm	Wrist			
SBP ≥ 135 mmHg	28.1%	47.1%	<0.0001	79.3%	0.58 ± 0.08*
DBP ≥ 85 mmHg	35.5%	56.6%	<0.0001	77.7%	0.57 ± 0.08*

*SBP* systolic blood pressure, *DBP* diastolic blood pressure. * *P* < 0.0001

## Discussion

Our study in a home-like setup settings reveals clinically significant differences between two validated HBPM devices. Notably, the wrist device identified nearly twice as many individuals as hypertensive for SBP compared to the upper-arm device. Casiglia et al, found similar results in large cohort tested in the office and at home with two other blood pressure devices [9]. This finding suggests that a person’s hypertension status could vary solely based on the device used, highlighting potential inconsistencies in BP classification across different devices. This raises important questions regarding measurement accuracy and the reliability of home BP data when used by patients and their healthcare provide in the management of hypertension. This aspect has not yet been evaluated in standard validation studies of commercial HBPM devices. This leads to two critical questions: which has a greater impact, the intrinsic accuracy of a BP monitor when tested in ideal conditions, or how patients use it? Additionally, what level of accuracy and precision should we ask for emerging technologies that could improve usability, knowing that existing technologies exhibit suboptimal precision and bias when used in home-like settings?

Cuff-based BP monitors, long considered the gold standard, are increasingly recognised as uncomfortable, unreliable, and inadequate for patient engagement, possibly contributing to the persistently low global hypertension control rates. Despite efforts to improve patient education and adherence to guidelines of correct HBPM usage, these devices fail to meet the demands of modern BP management. As a result, both patients and clinicians are trapped in a *status quo*, unable to rely on traditional home monitoring methods yet lacking access to better alternatives. This situation possibly perpetuates clinical inertia, as current methods are accepted despite their inadequacies. To break this cycle, we need new BP monitoring technologies that are automated, user-friendly, passive, continual, and capable of providing accurate data when used in real-world conditions. These advancements could not only improve patient comfort and engagement, but also enhance adherence, leading to better hypertension control. A possible limitation in our study was the absence of office blood pressure measurement that would have added insight of the setting effect. However, the objective in our study was to compare the device in a home-like setting. The absence of sensor may have introduced a bias through incorrect positioning of the wrist device. However, participants were provided with an instruction manual clearly showing the correct position. We did not record the error of position during the study.

Considering the relevant differences among validated blood pressure devices, measurements should be cross-checked and thoughtfully leveraged to ensure accurate and reliable clinical decisions. Finally, as the field of BP monitoring evolves, this study underscores the need for more guidance and education and critical analysis of home blood pressure data when used real-world conditions.

## Supplementary information

Supplementary Fig. 1

Supplementary Fig. 2

Supplementary Figures legends
