# Supplementary figures and images for: Comparison of two validated oscillometric devices in a home-like setup reveals pronounced blood pressure differences and reduced precision
Source: Hypertens Res. 2026 Jan 9;49(3):969–73. doi: 10.1038/s41440-025-02514-3 (PMC12960236; doi:10.1038/s41440-025-02514-3)

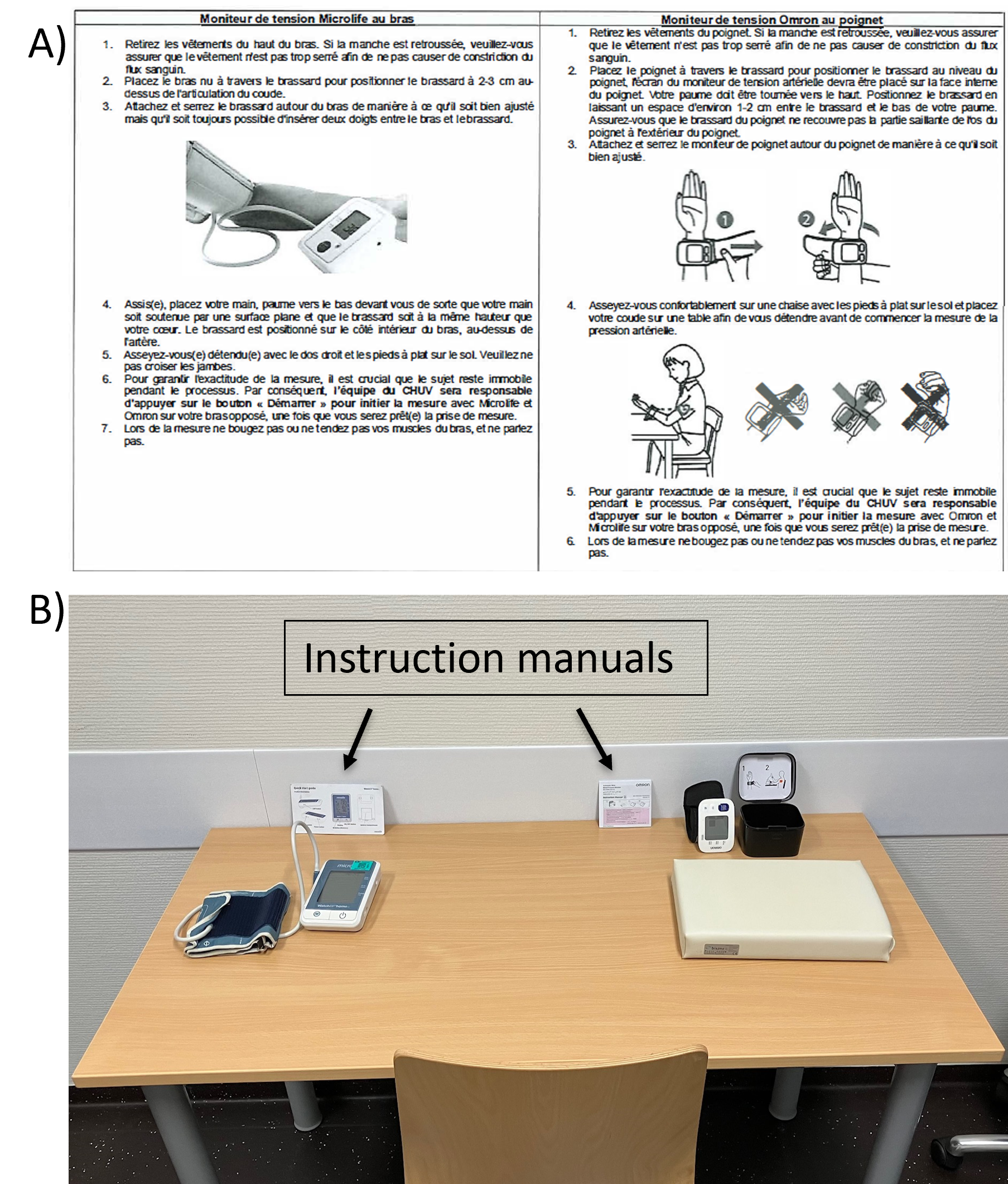

Supplement: Supplementary file 1 — Supplementary Fig. 1 [file 41440_2025_2514_MOESM1_ESM.tif]

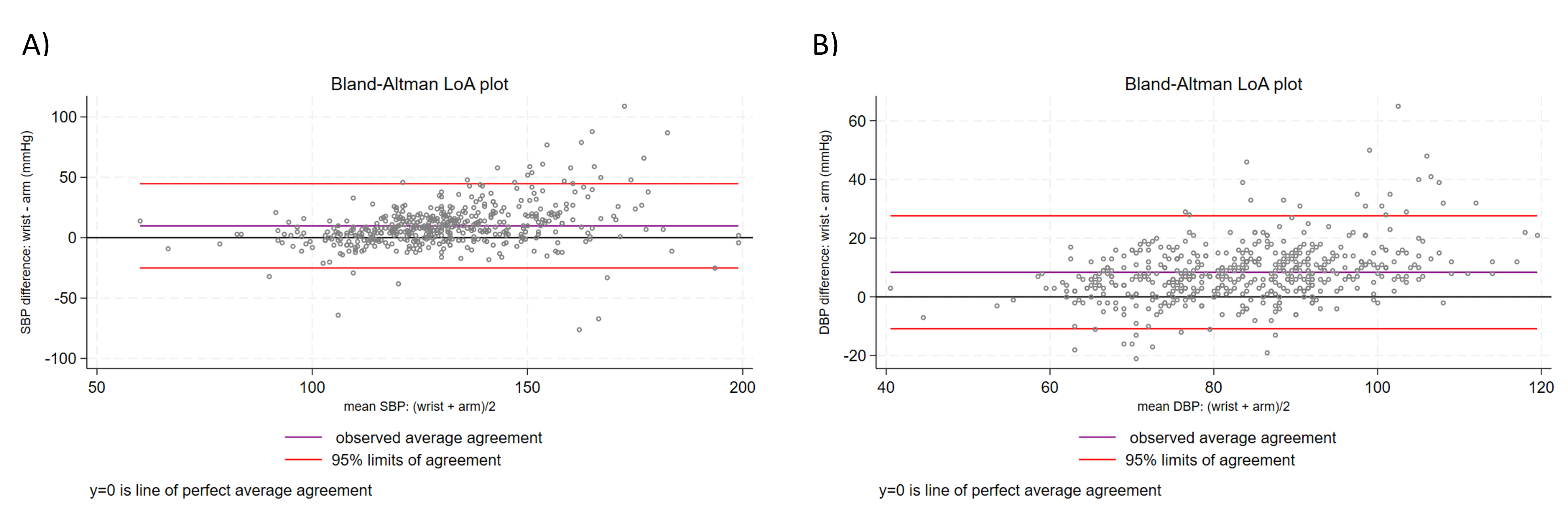

Supplement: Supplementary file 2 — Supplementary Fig. 2 [file 41440_2025_2514_MOESM2_ESM.tif]
