# Supplementary material for: Comparison of two validated oscillometric devices in a home-like setup reveals pronounced blood pressure differences and reduced precision
Source: Hypertens Res. 2026 Jan 9;49(3):969–73. doi: 10.1038/s41440-025-02514-3 (PMC12960236; doi:10.1038/s41440-025-02514-3)
Supplement: Supplementary file 3 — Supplementary Figures legends [file 41440_2025_2514_MOESM3_ESM.docx]

**Supplemental Figure 1**. Instructions and study setting. A) Detailed instruction of measurement of the upper-arm and the wrist device (in french) for participants. C) Picture of the study setting with both devices, instruction manuals and cushion for support.

**Supplemental Figure 2.** Bland and Altman plot of A) Systolic blood pressure (SBP) and B) Diastolic blood pressure (DBP). LoA: limits of agreement.
